# Extensive Peritonectomy is an Independent Risk Factor for Cisplatin HIPEC-Induced Acute Kidney Injury

**DOI:** 10.1245/s10434-022-12661-3

**Published:** 2022-12-10

**Authors:** Lukas F. Liesenfeld, Eva Quiring, Mohammed Al-Saeedi, Christian Nusshag, Markus W. Büchler, Martin Schneider

**Affiliations:** 1 Department of General, Visceral and Transplantation Surgery, Medical Faculty, University Hospital Heidelberg, Heidelberg, Germany; 2 Department of Nephrology, Medical Faculty, University Hospital Heidelberg, Heidelberg, Germany

## Abstract

**Background:**

Cisplatin (CDDP)-containing hyperthermic intraperitoneal chemotherapy (HIPEC) is frequently applied in selected patients with peritoneal malignancies derived from ovarian cancer, gastric cancer, and primary peritoneal mesothelioma. HIPEC with CDDP increases perioperative morbidity, in particular by inducing acute kidney injury (AKI). Factors contributing to occurrence of AKI after intraperitoneal perfusion with CDDP have not been sufficiently evaluated.

**Patients and Methods:**

Data from 63 patients treated with a CDDP-containing HIPEC regimen were retrospectively analyzed concerning demographics, underlying disease, surgery, and HIPEC details to evaluate risk factors of AKI. A preclinical rat perfusion model was applied to assess the influence of temperature, concentration, perfusate volume, perfusion flow rate, and extent of peritonectomy on drug absorption upon intraperitoneal CDDP perfusion.

**Results:**

AKI occurred in 66.1% of patients undergoing CDDP-containing HIPEC, with total intraoperative fluid influx being a negative and the extent of parietal peritonectomy being a positive independent predictor of postoperative AKI. In a preclinical model, bilateral anterior parietal peritonectomy significantly increased systemic CDDP absorption by 1.6 to 2-fold. CDDP plasma levels in animals were significantly higher after both perfusion with increased CDDP perfusate concentrations and bilateral anterior parietal peritonectomy.

**Conclusion:**

CDDP-containing HIPEC is associated with relevant morbidity owing to its systemic toxicity. Extent of parietal peritonectomy is an independent predictor of AKI. CDDP dose reduction should be considered in case of extensive parietal peritonectomy. Cytostatic drug concentrations in HIPEC perfusate should be paid more attention to than total dose per body surface area. Further clinical studies are needed to confirm the presented preclinical findings.

**Supplementary Information:**

The online version contains supplementary material available at 10.1245/s10434-022-12661-3.

Cytoreductive surgery (CRS) combined with hyperthermic intraperitoneal chemotherapy (HIPEC) has become a standard treatment for selected patients with peritoneal carcinomatosis (PC) of various origins.^1–3^ Cisplatin (CDDP)-containing HIPEC regimens are frequently applied in patients with peritoneal malignancies of ovarian and gastric origin, as well as in primary peritoneal mesothelioma.^4–6^ HIPEC in general is associated with increased perioperative morbidity.^7^ Particularly patients treated with CDDP-containing regimen encounter a high incidence of acute kidney injury (AKI).^8–11^ Reported incidence of HIPEC-induced AKI varies between 1 and 48%.^8, 12, 13^ Whether renal injury is induced by systemic nephrotoxic side effects of CDDP or by hyperthermia- and cytokine-induced vasodilatation of splanchnic vessels, resulting in relative hypovolemia and prerenal injury, is controversially discussed.^14–16^ Varying prevalence of AKI in different patient cohorts treated with CDDP-containing HIPEC, as well as prior pharmacokinetic studies, are questioning a cisplatin-related pathogenesis.^10, 12, 17, 18^ Clinical studies identified the use of CDDP in HIPEC regimen as a major risk factor for occurrence of HIPEC-induced AKI.^19–21^ In a preclinical model, perfusion with cisplatin was associated with high rates of AKI, whereas hyperthermic perfusion alone did neither induce AKI nor increase the incidence or severity of AKI when combined with cisplatin perfusion.^11^ Hitherto unconsidered factors, modifying cisplatin absorption from the intraperitoneal space, could explain divergent study results.

We have previously reported the incidence and risk factors for HIPEC-induced AKI in our patient cohort, identifying perfusion with cisplatin as the chief contributor to HIPEC-associated postoperative renal injury.^11^ The aim of the present follow-up study was to decipher specific risk factors for CDDP HIPEC-induced AKI, and to determine underlying factors modifying systemic CDDP uptake. For this purpose, we reanalyzed and extended clinical data from the subgroup of patients treated with CDDP-containing HIPEC. To specifically evaluate the influence of concentration, perfusate volume, perfusion flow rate, temperature, and extent of peritonectomy on systemic CDDP absorption, we applied a preclinical animal model of peritonectomy and HIPEC.

## Patients and Methods

### Study Design, Patient Population, and Data Source

Patients treated with CDDP-containing HIPEC between 2007 and 2020 at the Department of General, Visceral and Transplantation Surgery of the Heidelberg University Hospital were included in the present study. Data from 51 patients represented in this study were likewise included in our previous work reporting on general incidence and risk factors of HIPEC-induced kidney injury in 157 patients.^11^ According to the aims of our present follow-up study, novel data concerning surgical and HIPEC details were extracted from this group of patients, and data from 12 additional patients were incorporated. Patients were divided into two groups depending on the presence (AKI+) and absence (AKI−) of postoperative AKI. This study was approved by the local ethics committee of the Medical Faculty Heidelberg.

### Cytoreductive Surgery, Extent of Parietal Peritonectomy, HIPEC

All patients underwent exploratory laparotomy for assessment of peritoneal cancer index (PCI) and CRS if peritoneal nodules were present. HIPEC with or without early postoperative intraperitoneal chemotherapy (EPIC) was performed if a completeness of cytoreduction score ≤ 1 could be achieved, and patients’ PCI was ≤ 19.^22^ Closed-abdomen HIPEC was performed utilizing two inflow and two to three outflow drains with attached thermal probes. A PERFORMER-HT (RanD, Medolla, Italy) peristaltic pump was used for perfusion with an inflow temperature of 41–43 °C. Prior to 2019, a modified heart–lung machine (HLM) was utilized. Perfusate volume was set to 5000 ml or varied according to abdominal cavity volume if perfusion was performed with HLM.

Four distinct HIPEC regimens were applied, of which one was followed by EPIC with Taxol (TAX). One patient underwent HIPEC perfusion with CDDP mono (75 mg/m^2^), 27 patients with CDDP (50 mg/m^2^) and doxorubicin (DOX; 15 mg/m^2^) combined, 18 patients with CDDP (75 mg/m^2^) and mitomycin C (30 mg/m^2^) combined, and 10 patients underwent HIPEC with CDDP (50 mg/m^2^) and DOX (15 mg/m^2^) combined, followed by EPIC with Taxol (TAX; 80 mg/m^2^). Duration of HIPEC perfusion was 90 min, and HIPEC temperature was 42 °C in all applied regimens. EPIC treatment was performed daily for 5 days postoperatively.

**Fig. 1:**
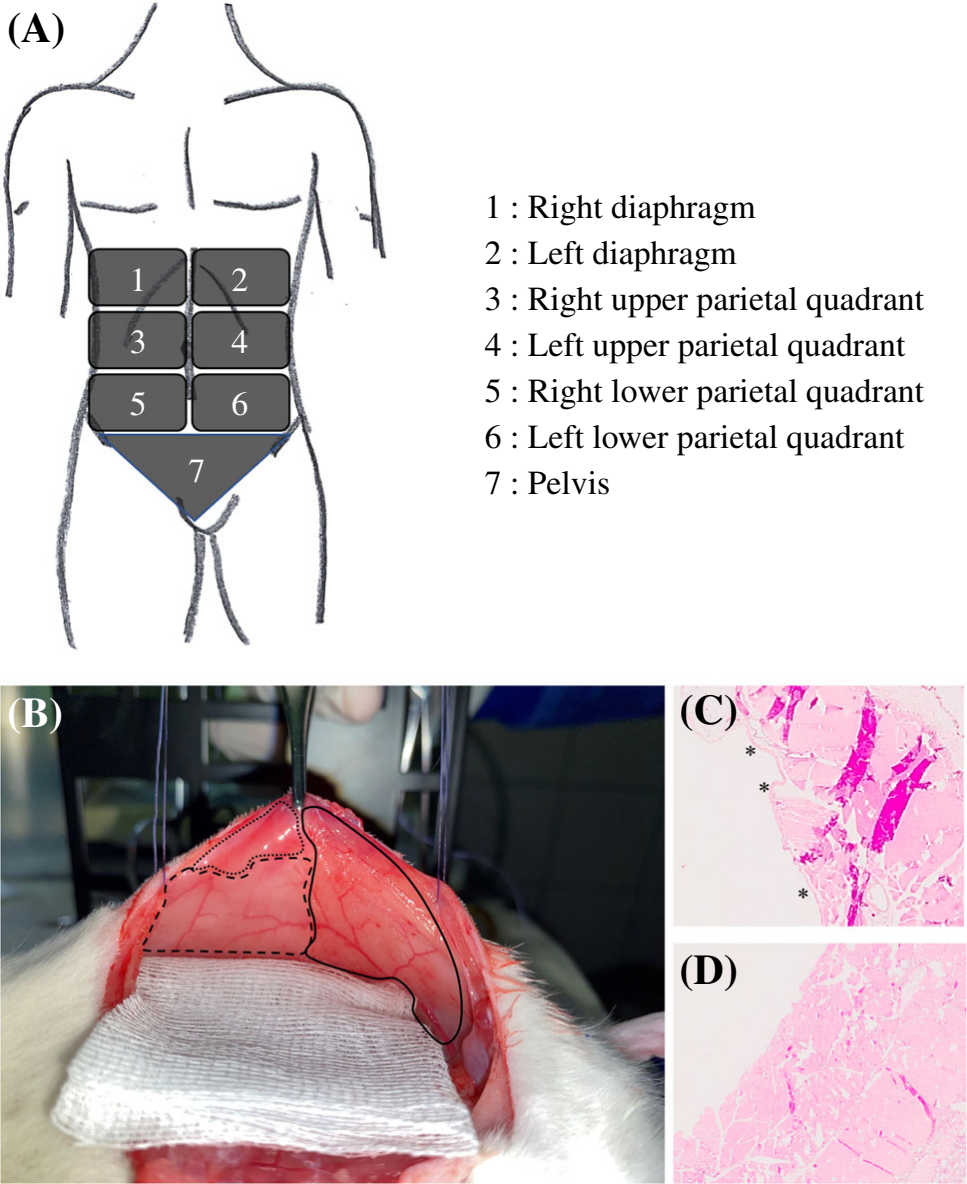
Parietal peritonectomy score (PPS) (**A**) and peritonectomy applying hydrodissection technique in rats (**B**–**D**). **A** Parietal peritoneum was divided in seven areas to quantify the extent of peritonectomy in human patients. Each area was assigned 1 point to a maximum score of 7 points (total parietal peritonectomy). **B** Left anterior abdominal wall with native peritoneum (dashed line; ---) and injected (hydrodissected) peritoneum before (dotted line; ···) and after peritonectomy (continuous line; ---). Corresponding hematoxylin-eosin stains before (**C**) and after (**D**) peritonectomy *peritoneal layer

The extent of parietal peritonectomy was retrospectively scored reviewing surgical reports (Parietal Peritonectomy Score; PPS) dividing the parietal peritoneum into seven distinct areas with a minimum score of 0 (no peritonectomy) to 7 points (total parietal peritonectomy) (Fig. 1A).

### Acute Kidney Injury

Serum creatinine levels were evaluated until postoperative day (POD) 10 and urine output until POD 2 to identify patients with AKI according to Kidney Disease Improving Global Outcomes (KDIGO) criteria.^23^ Fluid balance was calculated on the day of surgery as well as on POD 1 and 2, considering intravenous intake, oral intake and output (nasogastric tube), urine output, blood loss, and drains volume. A restrictive perioperative fluid strategy was commonly applied in CRS-HIPEC patients. Renal protectants or preoperative hydration strategies were not routinely applied.

### Animals

White (albino) CD (Sprague-Dawley) IGS rats (Crl:CD(SD)) were procured from Charles River (Sulzfeld, Germany) and housed in a specified pathogen free environment on a 12 h/12 h light/dark cycle with drink and feed ad libitum in accordance with institutional and federal animal welfare regulations. All experiments were approved by the responsible Karlsruhe Regional Council, Germany.

### Animal Model

HIPEC was performed applying a closed circuit using a peristaltic pump (Infusomat fmS, B. Braun Melsungen AG, Germany) and a water bath (Witeg WB-6, Wertheim, Germany) under isoflurane anesthesia.^24^ The body weight of animals varied between 210 and 250 g, body surface area was calculated using Meeh’s formula with *k* = 9.8.^25^ Perfusate volume was 1 or 2 L/m^2^ with 0.9% saline as perfusate solution. Flow rate was set to 20% or 10% of total perfusate volume/min. CDDP (37.5, 75, or 150 mg/m^2^; prepared at the Heidelberg University Hospital pharmaceutical department) was added when the desired temperature was reached, and perfusion was performed for 90 min. HIPEC inflow temperature was 38 °C (normothermia) or 42–43 °C (hyperthermia). Both, blood (500 µl each point in time) and perfusate (300 µl each point in time), were withdrawn 30, 60, 90, and 120 min after start of cisplatin perfusion. Transperitoneal cisplatin absorption rate was calculated by dividing CDDP concentration in plasma by CDDP concentration in perfusate at the same points in time after perfusion initiation. Blood samples were withdrawn from the retrobulbar venous plexus and (or) tail vein. Final samples were withdrawn via cardiac puncture. All blood samples were centrifuged immediately after withdrawal with plasma being stored at 4 °C. Abdomina were preloaded with 15 ml of 0.9% saline prior to the start of perfusion. At the end of perfusion, perfusate was aspirated from the peritoneal space and the abdomen was rinsed with 0.9% saline. Intraabdominal pressure (IAP) was measured invasively utilizing a piezoelectric pressure transducer (LogiCal, Smiths Medical, MN, USA). IAP was 7 ± 1 mmHg in all experimental groups.

In animals undergoing peritonectomy, parietal peritoneum of the center abdominal region and both flanks were resected (bilateral anterior parietal peritonectomy; equivalent to PPS = 4). For this purpose, small amounts of saline (0.9%) were injected underneath the peritoneal layer to dissect it off the abdominal wall muscles (hydrodissection technique, Fig. 1B). Tissues were fixed in 4% PFA (Otto Fischar GmbH & Co. KG, Germany) and embedded in paraffin (Fig. 1C, D). Experimental groups are listed and specified in Table 1.

**Table 1:** Experimental groups

Experimental group name	CDDP (mg/m^2^)	Temperature (°C)	Peritonectomy^#^	Perfusate volume (L/m^2^)	Flow rate (%perfusate vol/min)
Hyperthermia (reference group)	75	42	No	2	20
Concentration	**150**	42	No	2	20
Normothermia	75	**38**	No	2	20
Peritonectomy^#^	75	42	**Yes**	2	20
Perfusate volume	**37.5**	42	No	**1**	20
Perfusion flow rate	75	42	No	2	**10**

*CDDP* Cisplatin, ^#^bilateral anterior parietal peritonectomy, *n* = 8 for each group, parameters in bold are different to reference group (hyperthermia)

### Quadrupole Inductively Coupled Plasma Mass Spectrometry

All samples were processed within 4 weeks after withdrawal. Measurements were done by Dr. Limbach’s laboratory (Heidelberg, Germany) using quadrupole inductively coupled plasma mass spectrometry (ICP-MS). Platinum concentrations were assayed using a Thermo-Fisher Scientific iCap-RQ ICP-MS in standard mode. Samples were introduced from an autosampler (ESI SC-2) with 4 × 60-place sample racks; each sample loop was filled with 2 ml measuring solution through a concentric Mira Mist nebulizer (Burgener; 1 ml/min) and Peltier-cooled spray chamber (3 °C). Sample processing was undertaken using Plasmalab software (Thermo-Fisher Scientific). Quantification of platinum was carried out on the mass of the main isotope (Pt-195 u) of the element platinum.

### Statistical Analysis

Data are given as mean ± standard deviation unless otherwise specified. In normal distribution (according to the Kolmogorov-Smirnov), the student’s *t*-test was used when comparing two groups, otherwise regular one-way ANOVA was applied. If distributional requirements were not met or variables were dichotomous, the nonparametric Mann–Whitney *U* test or chi-squared test were used, respectively. Post-hoc analysis was performed using the Bonferroni-test. To evaluate the strength of association between variables, Phi coefficient/Cramer’s V was used if the independent variable was dichotomous, Spearman’s Rho when it was ordinal, and point-biserial correlation if it was continuously scaled. Binary multiple logistic regression was used to calculate the probability of AKI and assessment of model fit was done with Nagelkerkes *R*^2^ and Hosmer-Lemeshow test. Statistics were computed using IBM SPSS software (version 22.0; SPSS Inc., Chicago, IL). *p*-Values < 0.05 were considered significant.

## Results

### Patient and Tumor Characteristics

**Fig. 2:**
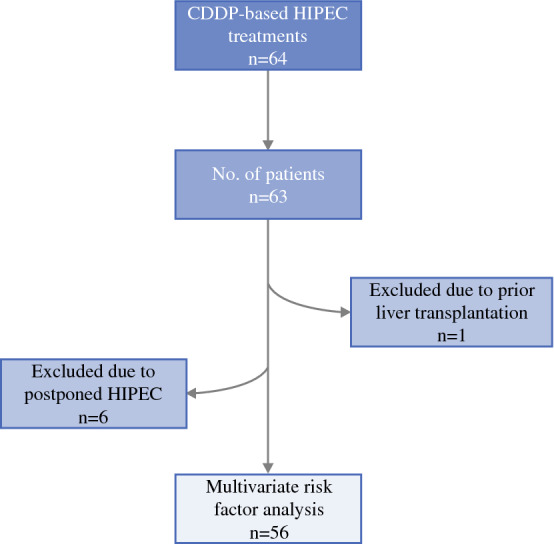
Flow chart diagram of the analyzed patient cohort. *CDDP* Cisplatin, *HIPEC* hyperthermic intraperitoneal chemotherapy, *CRS* cytoreductive surgery

Sixty-four CDDP-containing HIPEC’s (± EPIC) were performed in 63 patients (Fig. 2). One patient with recurrent mesothelioma was treated twice with HIPEC. Seven patients were excluded from renal injury risk factor analysis: in six patients HIPEC was postponed to few days after CRS and one patient underwent prior liver transplantation. Patient and tumor characteristics are presented in Table 2. There were slightly more female (*n* = 33, 58.9%) than male patients. Mean age was 61 ± 11 years and mean body mass index (BMI) was 25 ± 5.5 kg/m^2^. Preexisting chronic renal injuries or diabetes mellitus were not present in any of the patients. Twenty-six patients (48.1%) received neoadjuvant therapy prior to HIPEC treatment. Patients were treated with CDDP-containing HIPEC for mesothelioma (*n* = 17, 30.4%) and for PC from gastric (*n* = 24, 42.9%) or ovarian cancer (*n* = 15, 26.8%). HIPEC was performed with CDDP in one patient (1.8%), with CDDP and mitomycin C in 18 patients (32.1%), and with CDDP and doxorubicin in 27 patients (48.2%). In ten patients (17.9%), CDDP and doxorubicin HIPEC was combined with Taxol EPIC.

**Table 2:** Clinical data and univariate analysis of risk and protective factors for AKI after CDDP-containing HIPEC

Parameter	AKI+	AKI−	*p*-value	*R*
*N*	37 (66.1%)	19 (33.9%)		
Gender			0.1*	
Male	18 (32.1%)	5 (8.9%)		
Female	19 (33.9%)	14 (25%)		
Age (years)	61 ± 13	61 ± 10	0.97^#^	
BMI (kg/m^2^)	25 ± 5	25 ± 6	0.74^#^	
Congestive heart failure	1 (33%)	2 (66%)	0.22*	
Hypertension	9 (75%)	3 (25%)	0.46*	
ACE inhibitor	5 (55.5%)	4 (44.5%)	0.49*	
AT blocker	2 (100%)	0 (0%)	0.3*	
Diuretics	4 (100%)	0 (0%)	0.14*	
Primary tumor			0.77^^^	
Gastric	15 (63%)	9 (37%)		
Mesothelioma	11 (65%)	6 (35%)		
Ovarian	11 (73%)	4 (27%)		
Neoadjuvant CTx	17 (65.4%)	9 (34.6%)	0.91*	
CDDP-containing	1 (100%)	0 (0%)		
HIPEC regimen			0.47^^^	
CDDP + DOX	18 (66.7%)	9 (33.3%)		
CDDP + MMC	13 (72.2%)	5 (27.8%)		
CDDP	1 (100%)	0 (0%)		
CDDP + DOX + TAX	5 (50%)	5 (50%)		
Perfusate volume (ml)	3833 ± 1981	5217 ± 2320	0.056^#^	
CDDP concentration (µg/ml)	27.7 ± 9	22 ± 11.3	0.33^#^	
IO fluid (ml/kg/bw)	77 ± 31	106 ± 47	**0.017**^**#**^	− 0.343^##^
IO blood loss (ml)	1100 ± 1042	1089 ± 612	0.46^#^	
IO transfusion	7 (35%)	13 (65%)	< 0.001*	− 0.489**
IO vasopressors	31 (70.5%)	19 (29.5%)	0.063*	
IO SBP < 100 mmHg (min)	45 ± 40	34 ± 29	0.5^#^	
Duration of surgery (min)	464 ± 117	492 ± 158	0.82	
Fluid balance 1st POD (ml)	646 ± 1065	− 237 ± 1041	**0.02**^#^	0.362^##^
Fluid balance 2nd POD (ml)	− 487 ± 1553	142 ± 1118	0.33	
Vasopressors on 1st POD	10 (55.6%)	8 (44.4%)	0.36*	
Parietal peritonectomy score	4 ±6	2 ± 6	**0.04**^#^	0.277***
No. of resected organs^^^^	2 ± 1	3 ± 2	0.54^#^	

*HIPEC* hyperthermic intraperitoneal chemotherapy, *AKI* acute kidney injury, *BMI* body mass

index, *ACE* angiotensin-converting-enzyme, *AT* angiotensin, *IO* intraoperative, *CTx* chemotherapy, *CDDP* cisplatin, *DOX* doxorubicin, *MMC* mitomycin C, *TAX* Taxol *SBP* systolic blood pressure, *bw* body weight, *POD* postoperative day; ^#^according to Mann–Whitney *U* test; *according to chi-square test; ^according to ANOVA; **according to Phi-coefficient/Cramer’s V; ##according to point-biserial correlation; ***according to Spearman’s Rho; ^^excluding peritoneum; bold *= p < 0.05*

### Acute Kidney Injury After CDDP-Containing HIPEC

AKI, defined according to KDIGO criteria, occurred in 66.1% of patients (*n* = 37 out of 56 patients) treated with CDDP-containing HIPEC. Fifteen patients (40.5%) suffered stage 1, two patients (5.5%) stage 2, and 20 patients (54%) stage 3 renal injury. Five patients with stage 3 injury required temporary hemodialysis and two patients’ permanent renal replacement therapy. AKI did not occur concomitantly with septic complications in any patient. One patient was diagnosed with pneumonia at a point in time requiring temporary hemodialysis, but acute kidney injury was diagnosed 7 days beforehand. Demographic characteristics, underlying malignant disease and HIPEC regimen did not differ significantly in patients with and without renal injury (Table 2). Medical conditions predisposing for renal disease likewise did not differ between AKI+ and AKI− patients.

Data on the amount of HIPEC perfusate volume used were available for 21 patients. Though CDDP concentration in perfusate was higher in AKI+ patients (27.7 ± 9 versus 22 ± 11.3 µg/ml in AKI−) neither CDDP concentration nor perfusate volume correlated significantly with occurrence of AKI (*p* = 0.33 and *p* = 0.056, respectively).

Intraoperative fluid influx per body weight was lower in patients with AKI (77 ± 31 ml/kg/bw) than in those without AKI (106 ± 47 ml/kg/bw; *p* = 0.017, *r* = −0.343). Accumulated duration of intraoperative systolic blood pressure episodes < 100 mmHg was higher (45 ± 40 versus 34 ± 29 min; *p* = 0.5), and vasopressors were more frequently used in the AKI+ group (31 versus 19 patients, respectively; *p* = 0.063). Transfusion with packed red blood cells (pRBC) and/or fresh frozen plasma (FFP) negatively correlated with occurrence of postoperative AKI (*p* < 0.001; *r* = −0.489). Intraoperative blood loss was similar between the two groups (AKI+: 1100 ± 1042 ml; AKI−: 1089 ± 612 ml; *p* = 0.46).

Fluid balance on POD 1 was 646 ± 1065 ml in the AKI+ group and −237 ± 1041 ml in the AKI− group (*p* = 0.02; *r* = 0.362). On POD 2, fluid balance was −487 ± 1553 ml in the AKI+ group and 142 ± 1118 ml in the AKI− group (*p* = 0.33). On POD 1, 44.4% of patients without AKI and 55.6% of those with AKI received vasopressors (*p* = 0.36).

Parietal peritoneum was partially or completely resected in 42 cases (75%) (Supplementary Fig. 1). Total parietal peritonectomy (PPS = 7) was performed in 18 patients, and subtotal parietal peritonectomy (PPS = 6) was performed in three patients. Fourteen patients (25%) underwent CDDP HIPEC without concomitant resection of parietal peritoneum (PPS = 0). Extent of parietal peritonectomy was higher in AKI+ than in AKI− patients (4 ± 6 vs. 2 ± 6, respectively; *p* = 0.04), and positively correlated with the occurrence of postoperative AKI (*r* = 0.277). Number of resected visceral organs was 2 ± 1 in the AKI+ and 3 ± 2 in the AKI− group (*p* = 0.54; Supplementary Table 1). Duration of surgery did not differ between the two groups (AKI+: 464 ± 117 min; AKI−: 492 ± 158 min; *p* = 0.82).

Binary logistic regression confirmed intraoperative fluid influx (OR: 0.96) and extent of peritonectomy (OR: 1.52) to be independent negative and positive predictors of postoperative AKI, respectively (Table 3). To avoid multicollinearity, intraoperative transfusion was excluded from logistic regression analysis. The explained variance of the model was Nagelkerkes *R*^2^ = 0.43 and the Hosmer-Lemeshow test was not significant (*p* = 0.523).

**Table 3:** Binary multiple logistic regression of risk and protective factors for AKI after CDDP-containing HIPEC

Variable	OR	95% CI of OR	*p*-value
IO fluid influx	0.958	0.934–0.982	0.001
PPS	1.518	1.129–2.040	0.006

*AKI* acute kidney injury, *CDDP* cisplatin, *HIPEC* hyperthermic intraperitoneal chemotherapy, *IO* intraoperative, *PPS* parietal peritonectomy score, *OR* odds ratio, *CI* confidence interval

### Pharmacokinetics of Intraperitoneal CDDP perfusion

**Fig. 3:**
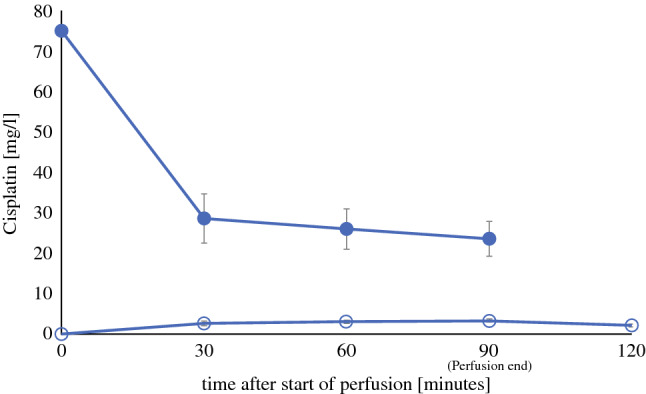
Pharmacokinetics of intraperitoneal CDDP perfusion in a preclinical rodent model. Time course of perfusate (full circles; *●*) and plasma CDDP concentrations (empty circles; *○*) in rats upon peritoneal perfusion with 150 mg/m^2^ CDDP in 2 L/m^2^ perfusate (75 mg/L CDDP in saline 0.9%) under hyperthermia (42–43 °C) conditions. Perfusion duration was 90 min at a flow rate of 20% perfusate vol/min. Data are expressed as mean ± SD (error bars); *n* = 5

A preclinical rat model of peritonectomy and HIPEC was applied to assess pharmacokinetic features of intraperitoneal cisplatin perfusion, and to further elucidate the significance of parietal peritonectomy in CDDP-HIPEC-associated AKI. Animals (*n = 5*) were treated with 150 mg/m^2^ CDDP for 90 min at 42 °C with 2 L/m^2^ perfusate volume (Fig. 3). Thirty min after start of perfusion, CDDP perfusate concentration dropped substantially to 38% (28.6 ± 6.1 mg/L) of the initial concentration (75 mg/L). Thereafter, concentration further declined to 26 ± 5 (35% of initial concentration) and 23.6 ± 4.3 mg/L (31% of initial concentration) at 60 and 90 min, respectively. CDDP concentration in plasma slowly but steadily rose to 2.7 ± 0.5, 3.1 ± 0.4, and 3.4 ± 0.4 mg/L at 30, 60, and 90 min, respectively. Thirty min after the end of perfusion, CDDP concentration decreased to 2.1 ± 0.3 mg/L.

### Systemic CDDP Absorption During Intraperitoneal Perfusion

Under conditions equal to those routinely applied in clinical practice, with 75 mg/m^2^ CDDP and 2 L/m^2^ perfusate volume at a temperature of 42–43 °C (Table 1: hyperthermia experimental group), CDDP absorption was 7.1 ± 2.3%, 9.2 ± 3.1%, and 11.1 ± 3.9% at 30, 60, and 90 min, respectively. This group served as a reference for further analyses.

**Fig. 4:**
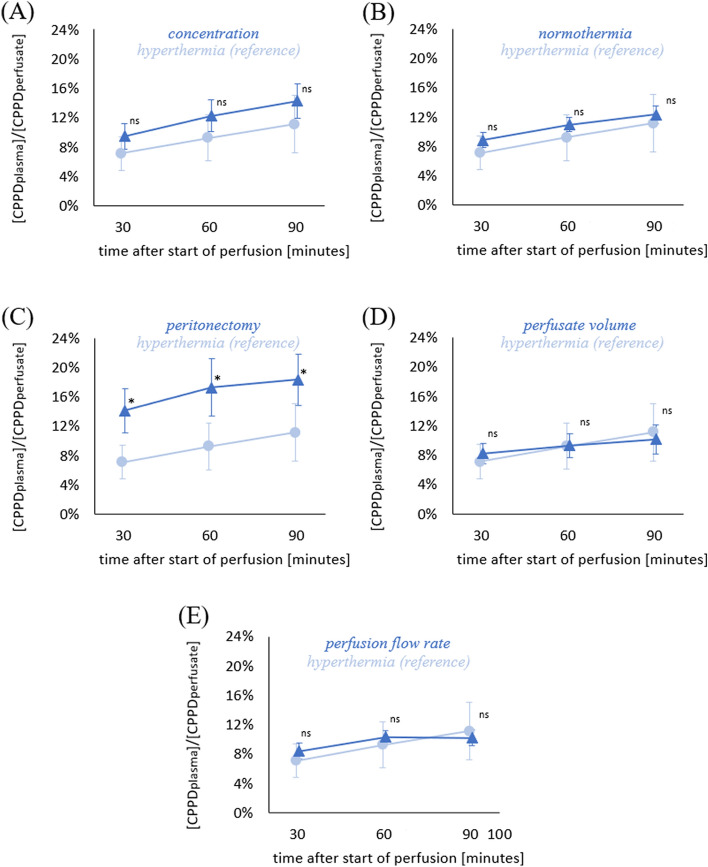
Impact of perfusate concentration, temperature, peritonectomy, perfusate volume, and perfusion flow rate on systemic CDDP absorption. Graphs reveal percentual amount of CDDP absorbed into plasma 30, 60, and 90 min after onset of intraperitoneal CDDP perfusion. The hyperthermia reference group (light blue circles) was perfused with a CDDP concentration of 75 mg/m^2^ at 21 L/m^2^ perfusate volume, a temperature of 42–43 °C, and a flow rate of 20% perfusate vol/min. Detailed information on experimental groups (blue triangles) with increased CDDP concentration (**A**), normothermic perfusion (**B**), parietal peritonectomy (**C**), reduced perfusate volume (**D**), or reduced perfusion flow rate (**E**) is provided in Table 1. Data are expressed as mean ± SD (error bars). *n* = 8 per experimental group; *p < 0.05; ^ns^*p* ≥ 0.05

Increasing the applied cisplatin dose to 150 mg/m^2^, yielding a higher CDDP perfusate concentration of 75 mg/L instead of 37.5 mg/L (Table 1: concentration experimental group), increased cisplatin absorption to 9.5 ± 1.7%, 12.2 ± 2.2%, and 14.3 ± 2.4%, respectively, but this difference did not reach statistical significance (Fig. 4A). Intraperitoneal CDDP perfusion under normothermia conditions (38 °C; Table 1: normothermia experimental group) did not alter cisplatin absorption compared with the hyperthermia reference group (Fig. 4B). However, bilateral anterior parietal peritonectomy (Table 1: peritonectomy experimental group) significantly increased cisplatin absorption to 14.1 ± 3%, 17.3 ± 3.9%, and 18.3 ± 3.5%, which is equivalent to a 1.6 to 2-fold increase compared with the hyperthermia group (*p* < 0.001; Fig. 4C).

To assess putative effects of perfusate volume or flow rate on CDDP absorption, perfusate volume was reduced to 1 L/m^2^ (resulting in a CDDP dose of 37.5 mg/m^2^; Table 1: perfusate volume experimental group), or flow rate was reduced to 10% of perfusate vol/min (Table 1: perfusion flow rate experimental group). Neither decreased perfusate volume nor decreased perfusion flow rate had any significant effects on CDDP absorption (Fig. 4D, E).

## Discussion

The currently observed broad diversity in utilized HIPEC regimens is a result of incomplete pharmacokinetic knowledge, which explains the heterogeneity of currently available data, and prompts skepticism concerning treatment benefits of additional HIPEC in patients undergoing cytoreductive surgery for treatment of peritoneal tumors.^26, 27^ Pharmacokinetic studies are, therefore, urgently required to identify and optimize relevant influential factors, thus improving outcomes of intraperitoneal perfusion therapy.

Owing to the drug’s cytotoxic efficacy in these diseases, HIPEC for peritoneal metastases of ovarian or gastric origin and for primary peritoneal mesothelioma is often performed with CDDP.^4, 28, 29^ In selected patients suffering these diagnoses, combining cytoreductive surgery with HIPEC provides promising oncologic outcomes but putatively elevated perioperative morbidity.^5, 9, 30, 31^ Prevalence of acute kidney injury after HIPEC is highly variable amongst various published patient cohorts.^12, 13^ However, previous work by others^18, 32^ and ourselves^11^ indicates that AKI is primarily induced by CDDP-derived nephrotoxicity. We therefore investigated specific risk factors contributing to acute kidney injury associated with CDDP HIPEC. Strikingly, in our patient collective, two thirds of all patients treated with CDDP-containing HIPEC developed acute kidney injury. Additional cytostatic drugs applied during HIPEC may have additionally contributed to renal injury; however, significant differences between different CDDP-containing HIPEC regimens could not be detected.

Among factors putatively affecting kidney function in patients undergoing CDDP HIPEC, Naffouje et al. identified blood loss as a major independent predictor of postoperative AKI.^16^ In our cohort, AKI occurred significantly less often when patients received intraoperative transfusion of blood components and had a high intraoperative fluid influx, with total intraoperative fluid influx being higher in transfused than in nontransfused patients. Transfusion positively correlated with intraoperative blood loss; however, blood loss did not differ between patients with or without renal injury. Vasopressors were more frequently used in patients with increased blood loss, however, their application per se did not correlate with the occurrence of AKI. Remarkably, intraoperative fluid therapy had nephroprotective properties in our cohort. Independently of replacement fluid type applied (crystalloid or colloidal). In contrast, Colantonio et al. found high intraoperative fluid influx to be accompanied with increased postoperative complication rates.^33^ Overall, currently available clinical data support the application of goal-directed fluid strategies, which reportedly have the potential to reduce postoperative complications and length of hospital stay in patients undergoing CRS and HIPEC.^33, 34^ Concerning postoperative fluid management, we found that fluid balance on POD 1 was significantly higher in patients suffering AKI than in those without. Neither use of vasopressors, nor intraoperative fluid influx, transfusion, blood loss, episodes with SBP < 100 mmHg, or urine output (all indicative for hypovolemic states and demand of fluid therapy), were significantly associated with fluid balance on POD 1. We therefore interpretate the difference in fluid balance between AKI− and AKI+ patients observed on POD 1 as a symptom and consequence of developing AKI, rather than as a causal factor.

Among other factors influencing pharmacokinetics, perfusate volume was lower and CDDP perfusate concentration was higher in patients developing AKI than in those without, although these differences did not reach statistical significance. Indeed, absorption of chemotherapeutics used in HIPEC increases with reduced volumes of perfusate,^35, 36^ a correlation that is not directly related to perfusate volume itself but to variance of drug concentrations in the perfusate.^37^ Importantly, the extent of parietal peritonectomy was significantly higher in patients developing AKI, and binary logistic regression confirmed the extent of peritonectomy to be an independent predictor of CDDP HIPEC-induced AKI. Currently available evidence for the influence of peritonectomy on HIPEC drug absorption is scarce, and the impact of peritonectomy on CDDP absorption during HIPEC has not previously been addressed experimentally. In our present collective of patients, the extent of parietal peritonectomy correlated with peritoneal disease severity, but not with the extent of visceral organ resections.

Remarkably, the prevalence of AKI induced by CDDP-containing HIPEC in our present patient cohort was about 15% higher than in previously reported patient series.^12^ We suspect that the prevalence of specific risk factors favoring CDDP HIPEC-induced AKI (such as the extent of parietal peritonectomy or CDDP perfusate concentration) was higher in this patient cohort than in previously published cohorts. In fact, previously published patient cohorts do not consistently provide information on the extent of parietal peritonectomy or CDDP perfusate concentration, which precludes appropriate comparison. However, we do not intend to rule out that the absence of routinely applied nephroprotective agents^38, 39^ or goal-directed fluid management strategies^34^ may have additionally contributed to a rather high incidence of CDDP HIPEC-induced AKI in our patient cohort.

We applied a preclinical rat model of HIPEC to specifically determine the impact of peritonectomy extent, along with other putatively relevant factors such as perfusate drug concentration, perfusate volume, temperature, and perfusion flow rate on systemic CDDP absorption. Remarkably, neither perfusion temperature, nor perfusate volume or perfusion flow rate had any influence on CDDP absorption. Bilateral anterior parietal peritonectomy, however, significantly increased CDDP absorption up to twofold dependent on the duration of perfusion, prompting significantly increased CDDP plasma concentrations. This finding emphasizes that the extent of peritonectomy is an independent risk factor for AKI owing to increased systemic renal CDDP toxicity. While conflicting data have been published on how the extent of peritonectomy affects the absorption of Mitomycin during HIPEC,^40, 41^ these preclinical data are consistent with previously reported clinical findings of increased Doxorubicin absorption in patients undergoing HIPEC along with extended parietal peritonectomy and visceral resection.^42^

The pharmacokinetics of intraperitoneal drug perfusion are complex, and factors affecting the depth of penetration and systemic absorption are not well defined yet. In the utilized animal model, a more substantial perfusate proportion is inside the tubing system than in the clinical setting, which may have relevant distributional effects. Moreover, absolute diffusion distances are relevantly shorter in rats than in humans, which could bias the effects observed in our preclinical animal model. We therefore neither intend to generally rule out any effects of temperature, perfusate volume, and perfusion flow rate on drug absorption, nor claim that the extent of peritonectomy is as relevant in patients as it is in our preclinical model.

Considering the currently available literature, perfusate drug concentration is a relevant factor affecting tissue penetration and systemic absorption.^28, 36, 37, 43^ In clinical practice, less a ttention has been dedicated to drug concentration in perfusate than to total drug dose, and perfusate volumes and consecutive drug concentrations are highly variable between studies.^27^ Not surprisingly, additional experiments carried out in the course of this study revealed that elevation of CDDP perfusate concentration affected the total amount of drug absorbed but not the absorption rate. This is consistent with findings previously reported by Royer et al., who observed a concentration-dependent occurrence of AKI in patients undergoing CDDP HIPEC for PC of ovarian origin. In their collective, 52% of patients treated with a perfusate concentration of 30 mg/L CDDP, but none of the 11 patients treated with concentrations of 15 mg/L CDDP developed AKI.^21, 44^ Conversely, Ansaloni et al. did not observe any nephrotoxic effects in patients undergoing HIPEC with CDDP concentrations of 43 mg/L CDDP.^15^ However, although perfusate concentrations were higher in the patient cohort reported by Ansaloni et al., maximum CDDP plasma concentrations were lower than in the cohort reported by Royer et al. Thus, while differences in CDDP plasma levels could explain the varying prevalence of nephrotoxicity observed in these studies, factors contributing to divergent CDDP plasma concentrations remain elusive.

In general, data on the extent of peritonectomy is rarely reported in the present literature on HIPEC efficacy and related toxicity, and a standardized classification system to assess it is missing. Precisely determining the extent of peritonectomy on the basis of retrospective clinical data collectives is therefore difficult, a limitation that partly applies to our present study as well. Heterogeneity of applied HIPEC drug regimens must also be considered. With respect to the ambiguous literature, the clinical findings and preclinical data provided in this study indicate that the extent of peritonectomy is indeed relevant concerning drug absorption and systemic side effects. Our data argue in favor of CDDP dose reduction in cases requiring extensive parietal peritonectomy. However, pharmacological studies and complementary prospective clinical studies, in which the extent of peritonectomy is precisely determined and correlated with toxicity profiles, are required to finally prove this conclusion.

## Conclusions

CDDP-containing HIPEC is commonly applied in selected patients with peritoneal malignancies but is associated with high nephrotoxicity. We show that increased intraoperative fluid influx is significantly accompanied by lower incidence of AKI, and that the extent of peritonectomy is an independent predictor of postoperative AKI in patients undergoing CDDP HIPEC. Consistently, bilateral anterior parietal peritonectomy significantly increased CDDP absorption in a preclinical animal model. Both increased concentration in perfusate and peritonectomy significantly increased CDDP plasma levels. Drug concentration in perfusate should be paid more attention to than total applied dose. Basic pharmacokinetic studies are needed to confirm the current findings and to identify further relevant influential factors.

## Supplementary Information

Below is the link to the electronic supplementary material.

Supplementary file1 (DOCX 13 KB)
