# Supplementary material for: Extensive Peritonectomy is an Independent Risk Factor for Cisplatin HIPEC-Induced Acute Kidney Injury
Source: Ann Surg Oncol. 2022 Dec 10;30(5):2646–56. doi: 10.1245/s10434-022-12661-3 (PMC10085927; doi:10.1245/s10434-022-12661-3)
Supplement: Supplementary file 1 — Supplementary file1 (DOCX 13 KB) [file 10434_2022_12661_MOESM1_ESM.docx]

**Supplemental Table 1: Quantity and extent of visceral organ resections.**

| Resected organ | Extent | No. |
| --- | --- | --- |
| Colon | (Sub-)total | 10 |
|  | (Extended) Hemicolon | 7 |
|  | Transversum | 1 |
|  | Sigmoid | 4 |
|  | Rectum | 9 |
| Intestine | Segment | 9 |
| Esophagus | Distal | 2 |
| Stomach | (Sub-)total | 14 |
|  | Partial | 1 |
| Liver | Segment | 1 |
|  | Atypical | 6 |
| Spleen | Total | 8 |
| Pancreas | Distal | 2 |
| Gallbladder |  | 27 |
| Omentum |  | 18 |
| Uterus |  | 5 |
| Adnexa | Unilateral | 2 |
|  | Bilateral | 6 |

**Supplemental Figure 1: *Grading of patients with (AKI+) and without (AKI-) acute kidney injury by extent of parietal peritonectomy utilizing the Parietal Peritonectomy Score (PPS).*** Parietal peritoneum was divided in seven distinct areas with a minimum score of 0 (no peritonectomy) to 7 points (total parietal peritonectomy).
